# Advising special population emergency medicine residency applicants: a survey of emergency medicine advisors and residency program leadership

**DOI:** 10.1186/s12909-020-02415-8

**Published:** 2020-12-07

**Authors:** Alexis E. Pelletier-Bui, Caitlin Schrepel, Liza Smith, Xiao Chi Zhang, Adam Kellogg, Mary Ann Edens, Christopher W. Jones, Emily Hillman

**Affiliations:** 1 Department of Emergency Medicine, Cooper Medical School of Rowan University, 1 Cooper Plaza, Camden, NJ 08103 USA; 2 Department of Emergency Medicine, University of Washington, 325 Ninth Avenue, Seattle, WA 98104 USA; 3 Department of Emergency Medicine, University of Massachusetts Medical School-Baystate, 759 Chestnut Street, Springfield, MA 01199 USA; 4 Department of Emergency Medicine, Thomas Jefferson University, 1020 Sansom St, 1651 Thompson Bldg, Philadelphia, PA USA; 5 Department of Emergency Medicine, Louisiana State University Health Sciences Center Shreveport, 1501 East Kings Highway, Shreveport, LA 71130 USA; 6 Department of Emergency Medicine, University of Missouri-Kansas City School of Medicine, 2411 Holmes Street, Kansas City, MO 64108 USA

**Keywords:** Medical student advising, Emergency medicine match, Applying for residency

## Abstract

**Background:**

The objective of this study was to determine the advising and emergency medicine (EM) residency selection practices for special population applicant groups for whom traditional advice may not apply.

**Methods:**

A survey was distributed on the Council of Residency Directors in EM and Clerkship Directors in EM Academy listservs. Multiple choice, Likert-type scale, and fill-in-the-blank questions addressed the average EM applicant and special population groups (osteopathic; international medical graduate (IMG); couples; at-risk; re-applicant; dual-accreditation applicant; and military). Percentages and 95% confidence intervals [CI] were calculated.

**Results:**

One hundred four surveys were completed. Of respondents involved in the interview process, 2 or more standardized letters of evaluation (SLOEs) were recommended for osteopathic (90.1% [95% CI 84–96]), IMG (82.5% [73–92]), dual-accreditation (46% [19–73]), and average applicants (48.5% [39–58]). Recommendations for numbers of residency applications to submit were 21–30 (50.5% [40.7–60.3]) for the average applicant, 31–40 (41.6% [31.3–51.8]) for osteopathic, and > 50 (50.9% [37.5–64.4]) for IMG. For below-average Step 1 performance, 56.0% [46.3–65.7] were more likely to interview with an average Step 2 score. 88.1% [81.8–94.4] will consider matching an EM-EM couple. The majority were more likely to interview a military applicant with similar competitiveness to a traditional applicant. Respondents felt the best option for re-applicants was to pursue the Supplemental Offer and Acceptance Program (SOAP) for a preliminary residency position.

**Conclusion:**

Advising and residency selection practices for special population applicants differ from those of traditional EM applicants. These data serve as an important foundation for advising these distinct applicant groups in ways that were previously only speculative. While respondents agree on many advising recommendations, outliers exist.

**Supplementary Information:**

The online version contains supplementary material available at 10.1186/s12909-020-02415-8.

## Background

Graduate medical education training programs in the United States (U.S.) utilize the National Resident Matching Program (NRMP) each year to “match” applicants from U.S. allopathic and osteopathic medical schools, and international medical schools in a fair, efficient, and reliable manner. After applicants apply and interview at residency programs, they create a rank order list of their preferred programs. Using a computerized mathematical algorithm, this list is aligned with program directors’ rank order lists of applicants in order to fill available training positions at U.S. teaching hospitals in a way that attempts to produce the best possible outcome for all stakeholders.

Advising increasingly heterogeneous emergency medicine (EM) applicants through this residency application process requires informed EM clerkship directors (CDs) and EM residency program leaders, including program directors (PDs), and assistant or associate program directors (APDs) [1, 2]. Advisors need to be familiar with the importance of the residency application components, including but not limited to: the U.S. Medical Licensing Exam (USMLE) Steps 1 & 2 Clinical Knowledge (CK) and Step 2 Clinical Skills (CS), which are required by U.S. allopathic and international medical graduates (IMGs) for licensure to practice medicine in the U.S. and optional for U.S. osteopathic applicants; the Comprehensive Osteopathic Medical Licensing Examination (COMLEX) Levels 1, 2-Cognitive Evaluation (CE) and 2-Performance Evaluation (PE), which are required for U.S. osteopathic licensure; and the standardized letter of evaluation (SLOE), the gold standard letter of evaluation that EM applicants traditionally obtain from each of their EM audition rotations at an academic institution associated with a U.S. EM residency program.

Existing EM student advising literature generally reflects U.S. allopathic seniors’ medical school experience [3–5]. Although the NRMP Data and Reports include dedicated publications for IMGs and osteopathic applicants to U.S. residency training programs, they do not address specific nuances in EM advising, such as the importance of the SLOE [6, 7]. Additionally, a recent study showed that osteopathic applicants find their mentorship and advising regarding SLOEs to be lacking, highlighting the need for quality advising for this growing applicant population to Accreditation Council for Graduate Medical Education (ACGME)-accredited residencies [8].

To address the changing applicant pool and need for expanded advising resources, the Council of Emergency Medicine Residency Directors’ (CORD) Advising Students Committee in EM (ASC-EM), a group of PDs, APDs, CDs and other advisors from EM residency programs across the country, developed evidence- and consensus-based advising resources for special population applicant groups and their advisors [9]. These groups include student cohorts which fall outside of the traditional allopathic medical school path to the NRMP Match, including: osteopathic students, IMGs, students who may be at risk of not matching, graduates looking to re-apply into EM, applicants to dual-accreditation programs (i.e. emergency medicine-internal medicine, or EM-IM), students linking their application with another student to pursue a couples match, and students pursuing a military match. Consensus-based advising publications in this context are increasing, but data to support published recommendations are lacking [10–13]. To inform evidence-based recommendations for medical student advising, this survey study seeks to define the advising and residency selection practices of CDs and EM residency program leaders for special population applicants.

## Methods

The CORD ASC-EM surveyed all individuals listed on the CORD and Clerkship Directors in EM (CDEM) Academy listservs using *Google Forms* online software. While exact listserv rosters are unavailable, in 2017 the Society of Academic Emergency Medicine (SAEM) clerkship directory listed 231 accredited EM residency programs and 167 EM clerkships [14, 15]. We sampled CDs, PDs, and APDs together, as they often serve dual roles in advising students and participating in residency interviewing and ranking. To encourage honest reporting practices and because advising practices can vary within individual residency programs, we did not collect program-identifying data. For data analysis, we inquired whether the respondents were involved in advising, interviewing/ranking or both. Respondents could choose multiple categories for title (CD, PD, APD, other faculty). The survey contained 56 questions in multiple choice, likert-type, and narrative format, divided into sections based on applicant-type: “average” (defined as high pass/honors grades, first-pass USMLE Step 1 score of 230, 1–2 scholarly projects, and no traditional red flags); osteopathic; IMG; couples; at-risk; re-applicant; dual-accreditation; and military (see Additional file 1). The average EM applicant was defined based on available NRMP match outcome data [16]. Questions assessed advising, residency interviewing, and residency ranking practices regarding each of these EM residency applicant populations.

The survey was reviewed by a survey methods expert (author CJ) and the CORD Board of Directors. It was piloted on 12 ASC-EM members (PDs, APDs, CDs) with experience in advising students and residency interviewing/ranking to ensure content validity and response process validity. The survey was then modified based on feedback and response latency for the entire survey.

We calculated proportions and 95% confidence intervals for the included categorical variables. Blank responses were removed from the denominator when calculating proportions. We compared point estimates and confidence intervals between EM residency program leaders and CDs for categorical responses. Data analysis was performed using Excel 2010 Version 14.0, Microsoft Corporation, Redmond, WA. The survey was administered over 4 weeks from December 2017 to January 2018. This survey was granted exempt status by the Institutional Review Board at the Cooper Health System.

## Results

### Survey respondents

The 104 medical educators who responded comprised CDs (29), PDs (40), APDs (33) and other faculty (9). Most were affiliated with allopathic programs (92/104), with 9 reporting affiliation with both allopathic and osteopathic programs and 1 reporting solely osteopathic affiliation. One reported no affiliation and 1 left this question blank. The majority advise EM-bound students (101/104) and participate in the EM residency interview process (101/104). For questions pertaining specifically to advising or ranking and interviewing applicants, only responses from those that reported being involved in these processes were used to analyze results. Only respondents who considered osteopathic and IMG applicants for ranking and matching at their program (*n* = 93 & 57, respectively) were asked follow-up questions regarding these applicant populations.

### Number of SLOEs and residency applications

For the average EM applicant, 79.2% [95% CI 71.3–87.1] of advisors involved in the EM residency interview process require 1 SLOE to grant an interview. Table 1 details the number of SLOEs recommended to rank average, osteopathic, IMG and dual-accreditation applicants. The number of residency applications recommended by respondents who advise medical students varied depending on applicant population (Table 2).

**Table 1:** Number of SLOEs suggested by respondents involved in the interview process according to applicant type

Applicant type (n)	Percent of respondents recommending number of SLOEs to rank an applicant [95% confidence interval]		
	1 SLOE	2 SLOEs	3 SLOEs
Average (101)	51.5 [41.8–61.2]	47.5 [37.8–57.2]	1 [0–2.9]
Osteopathic (91)	9.9 [3.8–16]	79.1 [70.8–87.5]	11 [4.6–17.4]
IMG (57)	17.5 [7.7–27.4]	63.2 [50.6–75.7]	19.3 [9.1–29.5]
Dual-accreditation (13)	54 [27–81]	46 [19–73]	0

*SLOEs* Standardized Letters of Evaluation, *IMG* International Medical Graduate

**Table 2:** Recommended number of ERAS applications, from respondents who advise medical students, according to applicant type

Applicant type (n)	Percent of respondents recommending number of ERAS applications [95% confidence interval]					
	1–10	11–20	21–30	31–40	41–50	> 50
Average (101)	2 [0–4.7]	14.9 [8–21.8]	50.5 [40.7–60.3]	23.8 [15.5–32.1]	7.9 [2.6–13.2]	1 [2.5–13.2]
Osteopathic (91)	2.2 [0–5.3]	6.7 [1.5–12.0]	16.9 [9.1–24.6]	41.6 [31.3–51.8]	20.2 [11.9–28.6]	12.4 [5.5–19.2]
IMG (57)	0 [0]	1.9 [0–5.5]	7.5 [0.4–14.7]	9.4 [1.6–17.3]	30.2 [17.8–42.5]	50.9 [37.5–64.4]
Couples match (101)	2 [0–4.8]	4.1 [1.7–8.0]	14.3 [7.4–21.2]	29.6 [20.6–38.6]	28.6 [19.7–37.5]	21.4 [13.3–29.5]

*ERAS* Electronic Residency Application Service, *IMG* International Medical Graduate

For some applicant types (e.g osteopathic students), CDs trended towards providing more conservative advice to residency applicants than EM residency program leaders (PDs, APDs), as demonstrated by recommendations to apply to a greater number of residency programs (Table 3). The limited sample sizes and wide confidence intervals limit our ability to conclusively evaluate this trend.

**Table 3:** Recommended number of ERAS applications, from CDs versus residency program leaders, according to applicant type

Applicant type	Respondent type (n)	Percent of respondents recommending number of ERAS applications [95% confidence interval]					
		1–10	11–20	21–30	31–40	41–50	> 50
Average	EM Residency Leader (72)	2.8 [0–6.6]	16.7 [8.1–25.3]	51.4 [39.9–62.9]	23.6 [13.8–33.4]	4.2 [0–8.8]	1.4 [0–4.1]
	EM Student Clerkship Director (29)	0	13.8 [1.2–26.4]	34.5 [17.2–51.8]	34.5 [17.2–51.8]	13.8 [1.2–26.4]	3.4 [0–10.0]
Osteopathic	EM Residency Leader (65)	3.1 [0–7.3]	6.2 [0.3–12.1]	20.0 [10.3–29.7]	38.5 [26.7–50.3]	23.1 [12.9–33.3]	9.2 [2.2–16.2]
	EM Student Clerkship Director (24)	0	8.3 [0–19.3]	8.3 [0–19.3]	45.8 [25.9–65.7]	8.3 [0–19.3]	29.2 [11.0–47.4]
IMG	EM Residency Leader (43)	0	2.3 [0–6.8]	9.3 [0.6–18.0]	9.3 [0.6–18.0]	32.6 [18.6–46.6]	46.5 [31.6–61.4]
	EM Student Clerkship Director (13)	0	0	7.7 [0–22.2]	7.7 [0–22.2]	23.1 [0.2–46.0]	61.5 [35.0–88.0]
Couples match	EM Residency Leader (71)	2.8 [0–6.6]	5.6 [0.3–10.9]	16.9 [8.2–25.6]	28.2 [17.7–38.7]	29.6 [19.0–40.2]	16.9 [8.2–25.6]
	EM Student Clerkship Director (27)	0	0	11.1 [0–22.9]	29.6 [12.4–46.8]	18.5 [3.9–33.1]	40.7 [22.2–59.2]

*ERAS* Electronic Residency Application Service, *IMG* International Medical Graduate, *CDs* clerkship directors, *EM* Emergency Medicine

### The “average” applicant

Only 7.9% [2.6–13.2] involved in the interview process reported USMLE Step 2 Clinical Knowledge (CK) as necessary for an interview of the “average” EM applicant. Most weigh Step 1 and Step 2 CK equally (49.5% [39.7–59.3]) or weigh Step 2 CK scores more than Step 1 scores (44.6% [34.9–54.3]).

### Special applicant populations

#### Osteopathic

Of those involved in the interview process (*n* = 91), 52.2% [42.0–62.4] will not consider applicants who have taken only the COMLEX, and not the USMLE. In contrast, 82.2% [74.3–90.1] will consider an osteopathic applicant who has only taken Step 1 and 57.3% [47.0–67.6] will consider an osteopathic applicant who has only taken Step 2 CK.

On thematic qualitative analysis of subjective responses, the most common responses for programs not ranking or matching osteopathic applicants (*n* = 10) were leadership decisions made at the medical school, departmental, or institutional level, uncertainty in evaluating the quality of training that osteopathic students receive, and a perception that matching osteopathic students would reflect poorly on their program.

#### The IMG applicant

**Fig. 1:**
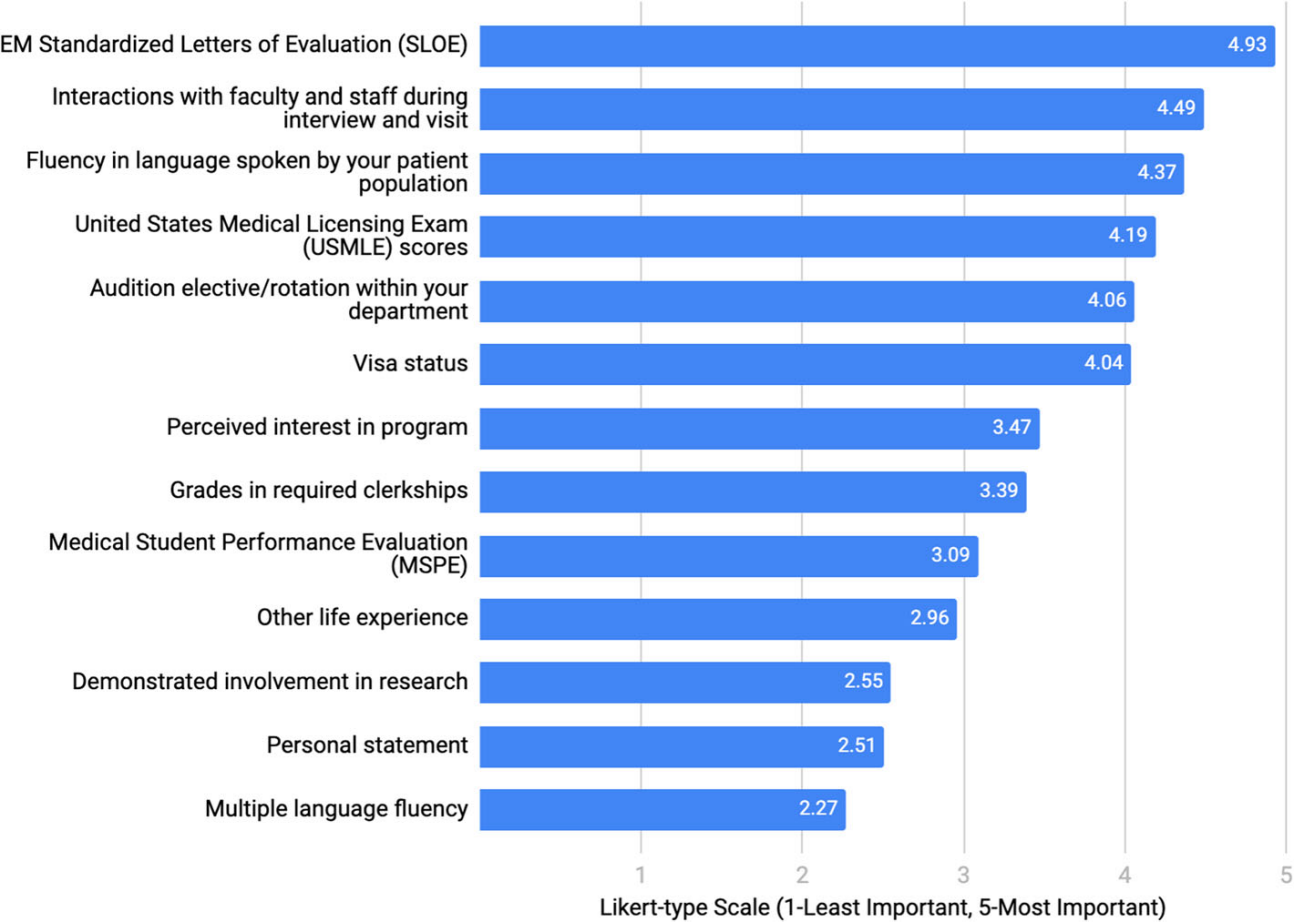
Factors of importance when considering an IMG applicant for an emergency medicine residency position (IMG: International Medical Graduate)

Regardless of other factors, 87.5% [78.8–96.2] of respondents recommended that IMGs apply to another specialty as back-up. Respondents ranked factors of importance (Fig. 1) when considering an IMG applicant for residency, using a 5-point Likert-type scale (5 being most important).

Thematic qualitative analysis of the subjective responses of those who do not consider IMGs for a residency position at their program (*n* = 44) revealed the following barriers: the already large number of allopathic U.S. applicants, unfamiliarity with the quality of the foreign educational process, departmental or institutional policies, and visa complications.

#### Couples match

Most respondents (80.2% [73.4–88.0]) attempt to coordinate interview dates for matching couples and will communicate with the PD of the other program regarding the matching couple (76.2% [67.9–84.5]). Many respondents (43.6% [33.9–53.2]) reported moving an applicant’s position on their rank list because they were matching with an applicant in another program. Also, 88.1% [81.8–94.4] report that their program will consider matching an EM-EM couple.

#### The at-risk applicant

Just over half of respondents involved in the interview process (53.5% [43.8–63.2]) had interviewed a student who had failed USMLE Step 1 in the past 3 years. In the past 3 years, the majority of respondents sometimes (5–15% of interviews) or frequently (> 15% of interviews) interviewed applicants with a below-average USMLE Step 1 (51.5% [41.7–61.1] and 35.6% [26.3–44.9], respectively). For applicants with a below-average USMLE Step 1 score, an available USMLE Step 2 CK score during initial application review increased the likelihood of respondents offering an interview, with 56.0% [46.3–65.7] more likely to interview with an average Step 2 CK score and 44.0% [34.3–53.7] with an above-average Step 2 CK.

Respondents involved in the interview process rated the frequency in the last 3 years with which they considered applicants with other common application red flags (Table 4).

**Table 4:** How often respondents interviewed applicants with red flags in the past 3 years

Red flag type	Percent of respondents interviewing applicants with red flags [95% confidence interval] *n* = 101		
	Never + Rarely (< 3 applicants/yr)	Sometimes (3–5 applicants/yr)	Frequently (> 5 applicants/yr)
Preclinical course failure	71.3 [62.5–80.1]	26.7 [18.1–35.3]	1 [0–2.9]
Clerkship failure	94.1 [89.5–98.7]	5 [0.7–9.3]	1 [0–2.9]
Criminal record	72 [63.2–80.8]	25 [16.5–33.5]	3 [0–6.3]
Unexplained gap in education	74 [65.4–82.6]	22 [13.9–30.1]	4 [0.2–7.8]
Academic misconduct	100	0	0

#### The re-applicant

**Fig. 2:**
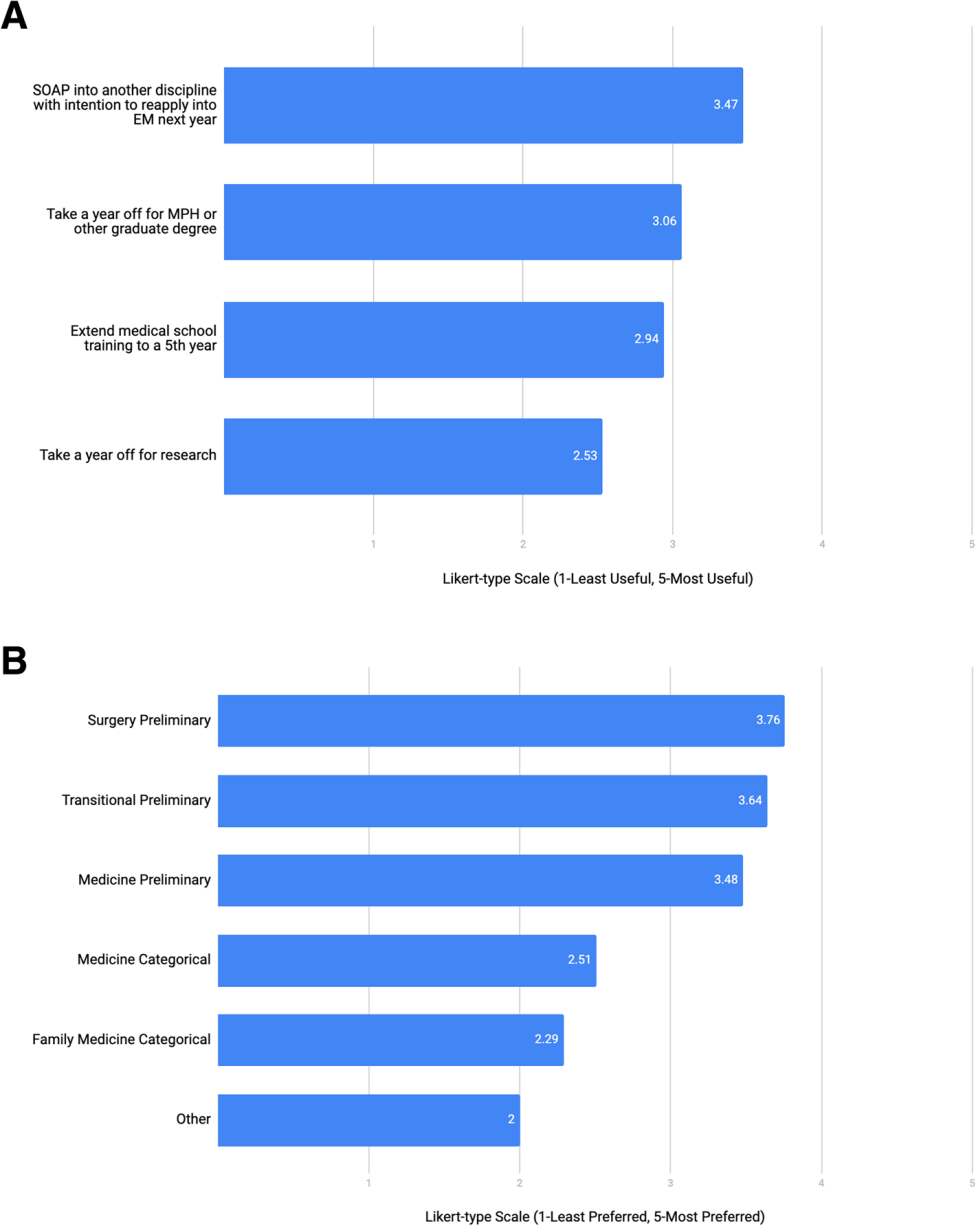
**a**. Best use of time during year before re-application cycle for applicant that does not match (SOAP: Supplemental Offer and Acceptance Program; MPH: Masters in Public Health; EM: emergency medicine). **b**. Preferred program type if an applicant pursues SOAP into another discipline after non-match in EM. (SOAP: Supplemental Offer and Acceptance Program; EM: emergency medicine)

When asked about advising applicants who plan to reenter the match to pursue an EM residency position, respondents rated different options for the year leading up to the re-application cycle as well as preferred disciplines through the Supplemental Offer and Acceptance Program (SOAP) (Fig. 2a and b).

Thematic qualitative analysis of the subjective responses indicated respondents had reservations advising students to take a categorical position with the intent of leaving after a year, considering the potential effect on the program. Respondents also underscored the recommendation that reapplicants pursue EM exposure in the interval to maintain skills and obtain additional recommendation letters.

#### Dual-Accreditation

Respondents involved in the interview process who have a categorical EM program only were asked how they view EM applicants who have also applied for a dual-accreditation program. Most stated it does not affect their ranking (82.4% [74.6–90.2]) while 17.6% [9.8–25.4] would be less likely to rank a dual applicant. All respondents with a dual-accreditation program (*n* = 13) reported considering applicants for both their dual-accreditation and categorical EM programs.

#### Military match

When asked to estimate how many students pursuing the military match they advise each year, 6 respondents advised more than 10 per year, whereas 77 respondents advised 3 or fewer per year. Only 26.0% [17.5–34.4] felt they understood the military match. The majority (53.1% [43.2–62.9]) did not know if they had the resources available to them to advise students through the military match process and 19.4% [11.6–27.2] reported they did not have any resources available. Many reported being more likely to interview a military applicant with similar competitiveness to a traditional applicant (average 3.55/5 on a Likert-type scale; 1-less to 5-more likely).

## Discussion

Our survey uncovered previously unreported data regarding advising and residency selection practices for special applicant groups. It provides data that can foster discussions about aligning how students are being advised with how students should be advised, enabling applicants and their advisors to approach the application process better informed.

### Standardized letter of evaluation

The SLOE is an important component of the EM-bound student’s residency application and directly impacts interview and ranking decisions [3, 5, 17]. Our study supports the available literature that the majority (> 95%) of PDs will accept 2 SLOEs for rank list placement [5, 18]. However, osteopathic and IMG students are often advised to obtain more SLOEs than the average applicant. Qualitative data suggests that requiring more SLOEs from these applicant groups may reduce perceived risk by assuaging concerns regarding the quality of training.

### Medical licensing examinations

A common reason applicants risk an unsuccessful match is below-average performance on medical licensing examinations [19]. We know that USMLE Step 2 CK is not required to grant an interview but is important for ranking [5, 14]. This study provides new insight into how the presence of a USMLE Step 2 CK score might affect a program’s willingness to extend an interview invitation to those who underperform on USMLE Step 1. For those that underperform on Step 1, performing well on USMLE Step 2 and taking the exam early enough for a score to be available when Electronic Residency Application Service (ERAS) opens is likely to increase their chances of obtaining a residency interview.

For the average EM applicant, our data also support at least one published study that found 95% of programs did not require USMLE Step 2 CK to grant interviews to average applicants [18]. In 2018, the NRMP PD survey showed that 51% of programs do not require USMLE Step 2 CK to grant an interview, and Negaard et al. reported 78% [3, 5]. Study methodology may explain the variable findings, for example, we defined the average applicant population as students with a Step 1 score equaling that of the average matched EM applicant (~ 230), which may account for respondents’ willingness to offer interviews. Our data regarding the weight that interviewers place on USMLE Step 1 versus Step 2 CK were similar to the 2018 NRMP PD survey, with USMLE Step 2 CK being preferred or viewed equally to Step 1 [3]. An important exception is that our data indicate that for osteopathic students who choose to take only one Step of the USMLE licensing exams, Step 1 is preferred. The reason for this preference is unclear.

Despite recently published data that demonstrate correlation between USMLE and COMLEX performance, our survey data show that osteopathic applicants are considered at the greatest number of ACGME programs if they have completed both USMLE Step 1 and Step 2 CK [20, 21]. Recently, the American Medical Association (AMA) approved Resolution 955 promoting equal acceptance of USMLE and COMLEX scores at all US residency training programs [22]. The effect this resolution and a single accreditation system will have on EM program preference for USMLE score reports is unclear. Qualitative comments indicate this preference for USMLE scores may be related to biases that extend beyond a simple acceptance of a single exam score as equivalent.

### Number of applications

Quality advising regarding the number of applications to submit can make a successful match more likely while also saving students time and money [23]. The 2019 NRMP applicant survey showed allopathic U.S. seniors who matched in EM applied to a median of 46 programs [24]. Meanwhile, the Association of American Medical Colleges (AAMC) reported 24 as the point of diminishing returns for the number of EM residency applications for allopathic US seniors with a USMLE Step 1 score of 219–234 [25]. Our results suggest that advisors may be contributing to the over-application problem among average EM-bound applicants. The AAMC provides no analysis for osteopathic, IMG, and couples match applicant groups with which to compare our data to determine if these recommended application numbers ideally balance match rate and unnecessary over-applying.

### Other considerations

Many special population applicants face barriers beyond their control in the residency application process. Discrimination against IMGs in the context of the residency application process has been published and our study confirms that expectations for osteopathic and IMG applicants varies from those of US MD applicants [26]. The number of respondents who do not consider osteopathic applicants for residency within their EM program is similar to previously reported [3]. According to our survey, uncertainty regarding how to evaluate these students’ performances outside of the SLOE and USMLE board scores affected how respondents considered osteopathic and IMG applicants. For osteopathic applicants it is unclear how the transition to a single accreditation system will affect the residency applicant screening and ranking practices.

While the aforementioned barriers may not be modifiable, the survey responses do offer some tangible suggestions for IMG and osteopathic applicants. They should focus on taking licensing examinations early and obtaining an audition elective at an institution where they would like to match. IMG applicants should strive to master the English language, as it was listed as a top reason to consider IMG applicants, and may want to spend less time on pursuing research. These results can also provide guidance for when to consider applying to another specialty as a backup plan.

According to the NRMP, U.S. senior couples match at the same rate as their peers [27]. When reviewing couples, programs make accommodations that can benefit applicants. Our respondents may adjust students’ position up or down on the rank list as a result of their couples status, and some programs will not consider EM-EM couples; thus, EM-EM couples may need to submit more residency applications.

It is important for an individual applying to dual programs to consider the impact of this revelation to the non dual-accreditation programs to which s/he is applying considering 17.6% stated they would be less likely to rank this applicant.

We found no prior data that suggest the best use of time following a non-match in EM. In the 2019 NRMP applicant survey, U.S. seniors in this position stated they would be most likely to participate in the SOAP, first in their preferred specialty, followed by a preliminary year position, then a less competitive backup specialty [24]. In our survey, we did not include the option to participate in the SOAP for EM given the scarcity of EM SOAP positions [27]. There is a discrepancy between U.S. seniors’ strategies following a non-match as reported in the 2019 NRMP applicant survey and our data. For example, unmatched U.S. seniors’ next highest rated strategy would be to pursue research and re-enter the match the following year, which our respondents reported to be the least beneficial use of time. Moreover, unmatched students were least likely to pursue a graduate degree, yet our respondents rated this the next most beneficial use of time. Awareness of these differences between advisors’ and students’ perspectives is important and can inform targeted efforts to align practices and recommendations.

Our data highlight numerous other potential areas where advisors can help their unmatched or at-risk students better strategize. For example, medical school course or clerkship failures, academic misconduct, and legal problems were significantly more disqualifying than board performance [3]. As such, applicants with these red flags should have a frank and early discussion with their advisor about application strategies and backup plans.

Military applicants are outliers among the special population groups, as respondents were more likely to interview those with similar competitiveness to a traditional applicant. The uncertainty of most respondents about the military match illustrates the importance of developing evidence-based advising resources for EM special applicant populations.

#### Limitations

Our respondents (104) compared to the total number of ACGME-accredited EM residency programs (231) was limited. The survey may have captured multiple respondents from individual institutions, further lowering the response rate; we did not apply a limit of 1 response per institution in order to encourage honest reporting and maintain blinding of the results. Also multiple people of differing roles (CDs, APDs, PDs) often contribute to both advising and the residency application process within a single institution; by limiting responses to just one of these roles, we would miss perspectives from important stakeholders in the advising and residency selection process. As a result, geographical variations in responses is not known. Also, those educators who chose to respond may differ from those who did not with respect to their advising and interviewing practices. We also recognize that although our data reflect current advising strategies, identifying the best strategy for a particular applicant requires an individualized approach.

## Conclusion

This survey adds to the existing literature by providing much-needed data to inform advising an increasingly diverse applicant population. The trends we identified suggest that evidence-based advising is much more nuanced than previously known, and those advising EM-bound students can leverage our findings to the maximal benefit of the students. This information is important for all stakeholders in the EM residency advising and interviewing processes.

## Supplementary Information

**Additional file 1.**

## Data Availability

The datasets used and/or analysed during the current study are available from the corresponding author on reasonable request.
