# Supplementary material for: Advising special population emergency medicine residency applicants: a survey of emergency medicine advisors and residency program leadership
Source: BMC Med Educ. 2020 Dec 7;20:495. doi: 10.1186/s12909-020-02415-8 (PMC7722429; doi:10.1186/s12909-020-02415-8)
Supplement: Supplementary file 1 — Additional file 1. [file 12909_2020_2415_MOESM1_ESM.docx]

**Section 1 of 14**

CORD SATF Advising Addenda Survey

The CORD Student Advising Task Force (SATF) aims to provide quality, consistent advising to students and advisors across the country. The goal of this survey is to clarify best recommendations for our emergency medicine-bound applicants and special population applicants. Your involvement in this survey is voluntary. By completing this IRB approved anonymous survey, you are consenting to participation. You may decide to withdraw your participation at any point during the survey by simply exiting the survey. There is no penalty for declining participation.

Demographics

1. What title(s)/positions(s) do you currently hold? (check boxes - choose all that apply)

- Clerkship Director
- Assistant Clerkship Director
- Program director
- Assistant/Associate Program Director
- Other

1. If you chose other, what is your title/position? (free text)
2. With what type of residency program are you affiliated? (choose one)

- Allopathic accredited
- Osteopathic accredited
- Both allopathic & osteopathic accredited
- I am not affiliated with a residency program

1. Do you advise emergency medicine-bound students? (choose one)

- Yes
- No

1. Are you involved in the emergency medicine residency interview process? (choose one)

- Yes
- No

*After section 1, continue to the next section*

**Section 2 of 14**

The “Average” Applicant

(ex: High pass/honors grades, First-pass USMLE Step 1 score of ~230, 1-2 scholarly projects & no red flags)

1. How many SLOEs do you require to grant an interview? (choose one)

- 1
- 2
- 3 or more

1. How many SLOEs do you require to rank an applicant? (choose one)

- 1
- 2
- 3 or more

1. Do you require USMLE Step 2 to offer an interview to an applicant? (choose one)

- Yes
- No

1. How do you treat USMLE Step 2 score in relation to USMLE Step 1 scores? (choose one)

- Take both into consideration equally when evaluating applicant
- Step 2 replaces step 1 when available (consideration only given to Step 2 score)
- Step 2 outweighs step 1 score significantly
- Step 1 outweighs step 2 score significantly

1. When advising the average EM applicant, how many applications do you suggest he/she submit? (choose one)

- 1-10
- 11-20
- 21-30
- 31-40
- 41-50
- >50

*After section 2, continue to the next section*

**Section 3 of 14**

Couples Match

1. Will your program attempt to coordinate interviews for matching couples? (choose one)

- Yes
- No

1. Do you talk to the PD of the other program regarding the matching couple? (choose one)

- Yes
- No

1. Have you ever moved a resident up or down on your rank list because they were matching with a resident in another program? (choose one)

- Yes
- No

1. Would you consider matching an EM-EM couple to your program? (choose one)

- Yes
- No

1. When advising the "average" EM applicant who intends to couples match with another "average" student (in any discipline), how many applications do you suggest he/she submit? ("average" example: High pass/honors grades, First-pass USMLE Step 1 score of ~230, 1-2 scholarly projects & no red flags) (choose one)

- 1-10
- 11-20
- 21-30
- 31-40
- 41-50
- >50

*After section 3, continue to the next section*

**Section 4 of 14**

At Risk Applicant

1. To the best of your knowledge, in the past 3 years has your program interviewed an applicant who has failed USMLE Step 1? (choose one)

- Yes
- No

1. If you consider applicants who have failed USMLE Step 1, what impact does repeat Step 1 have? (choose all that apply)

- I would consider applicant for interview if repeat Step 1 was below average (<230)
- I would consider applicant for interview if repeat Step 1 was average (~230)
- I would consider applicant for interview only if repeat Step 1 was above average (>230)
- Not applicable - I do not consider applicants who have failed Step 1

1. To the best of your knowledge, how many times in the past 3 years has your program interviewed a student who has a below average USMLE Step 1 score? For reference, average USMLE for matched EM applicants in 2016 was 233. (choose one)

- Never
- Rarely (<5% of interviews)
- Sometimes (5-15% of interviews)
- Frequently (>15% of interviews)

1. For applicants with a below average USMLE Step 1 score, would an early Step 2 score (score available when application submitted) increase your likelihood of interviewing? (choose one)

- I still would not/would rarely interview with a below average step 1 score
- More likely to interview if they have an average step 2 score
- Would only increase likelihood of interview if above average step 2 score

1. To the best of your knowledge, in the past 3 years how often has your program interviewed a student who failed a preclinical course? (choose one)

- Never
- Rarely (<3 applicants/year)
- Sometimes (3-5 applicants/year)
- Frequently (>5 applicants/year)

1. To the best of your knowledge, in the past 3 years how often has your program interviewed a student who failed a clerkship? (choose one)

- Never
- Rarely (<3 applicants/year)
- Sometimes (3-5 applicants/year)
- Frequently (>5 applicants/year)

1. To the best of your knowledge, in the past 3 years how often has your program interviewed a student who has a criminal record (ex: driving under the influence, drug possession or other)? (choose one)

- Never
- Rarely (<3 applicants/year)
- Sometimes (3-5 applicants/year)
- Frequently (>5 applicants/year)

1. To the best of your knowledge, in the past 3 years how often has your program interviewed a student who has an unexplained gap in education? (choose one)

- Never
- Rarely (<3 applicants/year)
- Sometimes (3-5 applicants/year)
- Frequently (>5 applicants/year)

1. To the best of your knowledge, in the past 3 years how often has your program interviewed a student with a history of academic misconduct? (choose one)

- Never
- Rarely (<3 applicants/year)
- Sometimes (3-5 applicants/year)
- Frequently (>5 applicants/year)

*After section 4, continue to the next section*

**Section 5 of 14**

The Re-Applicant

1. For an applicant that does not match, what do you consider the best use of time during the year leading up to/during the re-application cycle? Please rank the following factors in terms of value on a Likert scale (1-least valuable use of time to 5-most valuable use of time)
   - Take a year off for research (choose 1-5)
   - Take a year off for MPH or other graduate degree (choose 1-5)
   - Extend medical school training to a 5th year (choose 1-5)
   - SOAP into another discipline with the intention reapply into EM the following year (choose 1-5)
2. Are there other ways that you would like to see an applicant spend their time during the year of the re-application cycle? (free text)
3. If an applicant chooses to SOAP into another discipline (with intention to match in EM the next application cycle), which specialty or program type is preferred? Please rank the following factors in terms of value on a Likert scale (1-least valuable use of time to 5-most valuable use of time)
   - Transitional prelim (choose 1-5)
   - Surgery prelim (choose 1-5)
   - Medicine prelim (choose 1-5)
   - Medicine categorical (choose 1-5)
   - Family medicine categorical (choose 1-5)
   - Other
4. If you chose other, which other discipline would you prefer to see an applicant match into? (free text)

*After section 5, continue to the next section*

**Section 6 of 14**

The Osteopathic Applicant

1. Do you consider osteopathic (DO) applicants for a residency position at your program? (choose one)

- Yes > *Branch to section 8*
- No > *Branch to section 7*

**Section 7 of 14**

The Osteopathic Applicant

1. Why don't you consider osteopathic applicants for a residency position at your program? (free text)

*After section 7, go to section 9 (The Military Applicant)*

**Section 8 of 14**

The Osteopathic Applicant

1. Do you consider osteopathic applicants who have only taken the COMLEX and not the USMLE? (choose one)

- Yes
- No

1. Do you consider osteopathic applicants who have only taken USMLE Step 1? (choose one)

- Yes
- No

1. Do you consider osteopathic applicants who have only taken USMLE Step 2? (choose one)

- Yes
- No

1. For applicants who submit both COMLEX and USMLE scores, how much weight do you give the COMLEX? (choose one)

- Highly weighted
- Slighted weighted
- Only matters if there is a failure in the COMLEX score
- Not weighted at all

1. When considering an osteopathic student for a medical student rotation, do you require a USMLE Step 1 score? (choose one)

- Yes
- No

1. When advising an osteopathic applicant, how many SLOEs do you suggest he/she obtain? (choose one)

- 1
- 2
- 3 or more

1. When advising an "average" osteopathic applicant, how many applications do you suggest he/she submit to an allopathic residency program? ("average" example: High pass/honors grades, First-pass USMLE Step 1 score of ~230 or COMLEX score of ~600, 1-2 scholarly projects & no red flags) (choose one)

- 1-10
- 11-20
- 21-30
- 31-40
- 41-50
- >50

*After section 8, continue to the next section*

**Section 9 of 14**

The Military Applicant

1. Do you feel you understand the military match process? (choose one)

- Yes
- No
- Somewhat

1. On average, how many students do you have or advise going through this process each year? (free text)
2. Does your associated medical school accommodate the high priority need for multiple early away rotations for military applicants (i.e. flexible scheduling for these applicants)? (choose one)

- Yes
- No
- I don’t know

1. Do you have a resource list, military interest group, or avenue to direct students for further mentorship in this process? (check all that apply)

- Resource list
- Military interest group
- Military mentor
- We have no resources
- I don’t know (52)=53.1% (43.2-62.9)
- Other (free text option)

1. How likely are you to interview a military applicant with similar competitiveness to a traditional applicant? (Likert scale, 1 less likely-5 more likely)
2. When do you offer interviews to military applicants?

- Any time of the interview season
- Early in my interview season
- Late in my interview season
- Not applicable

*After section 9, continue to next section*

**Section 10 of 14**

The Dual Program Applicant

1. If you ONLY have a categorical EM program (not a dual program), how do you view an EM applicant who has also applied for a dual program (EM-IM, EM-FM, EM-Peds or EM-anesthesia)? (choose one)

- Less likely to rank
- No effect on my ranking
- More likely to rank
- N/A – I have a dual program

1. Do you have a combined EM program (EM-IM, EM-FM, EM-Peds or EM-Anesthesia)? (choose one)

- Yes > *Branch to next section (section 11)*
- No > *Branch to section 12 (IMG Applicants)*

**Section 11 of 14**

Dual Program Applicants

1. How many SLOEs do you require to grant an interview? (choose one)

- 1
- 2
- 3 or more

1. How many SLOEs do you require to rank an applicant? (choose one)

- 1
- 2
- 3 or more

1. Do you consider applicants for both your joint program and categorical EM program? (choose one)

- Yes
- No

1. Do you consider applicants for both your joint program and your other categorical program (IM, FM, Peds, Anesthesia)? (choose one)

- Yes
- No

*After section 11, continue to next section*

**Section 12 of 14**

The International Medical Graduate (IMG) Applicant

US-IMG: US citizen who attends medical school outside the US

Non-US IMG: Non-US citizen who attends medical school outside the US

US senior: Anyone who attends medical school within the US (US citizen or non-US citizen)

1. Do you consider IMG applicants for a residency position at your program?

- Yes *> Branch to section 14*
- No *> Branch to section 13*

**Section 13 of 14**

International Medical Graduate (IMG) Applicants

1. Why don't you consider IMG applicants for a residency position at your program? (free text)

*After section 13, submit form*

**Section 14 of 14**

International Medical Graduate (IMG) Applicants

1. Would you consider an IMG applicant who does not have a SLOE? (choose one)

- Yes
- No

1. How many SLOEs do you feel an IMG applicant should obtain? (assume application otherwise similar to the average non-IMG EM applicant) ("average" example: High pass/honors grades, First-pass USMLE Step 1 score of ~230, 1-2 scholarly projects & no red flags) (choose one)

- 1
- 2
- 3 or more

1. When advising an IMG applicant, how many applications do you recommend he/she submit? (assume application otherwise similar to the average non-IMG EM applicant) ("average" example: High pass/honors grades, First-pass USMLE Step 1 score of ~230, 1-2 scholarly projects & no red flags) (choose one)

- 1-10
- 11-20
- 21-30
- 31-40
- 41-50
- >50

1. When advising an IMG applicant do you recommend he/she apply to another speciality as back-up? (assume application otherwise similar to the average non-IMG EM applicant) (choose one)

- Yes
- No

1. When considering an IMG applicant for an EM residency position, please rank the following factors in terms of their importance to you on a Likert Scale (1-least important to 5-most important):

- USMLE scores
- EM Standardized Letters of Evaluation (SLOE)
- Medical Student Performance Evaluation
- Grades in required clerkships
- Demonstrated involvement in research
- Visa status
- Fluency in language spoken by your patient population
- Multiple language fluency
- Other life experience
- Personal Statement
- Audition elective/rotation within your department
- Interactions with faculty and staff during interview and visit
- Perceived interest in program

1. What concerns do you have when reviewing IMG applicants? Please rank the following factors in terms of their importance to you on a Likert Scale (1-least concerning to 5-most concerning)

- Insufficient English language proficiency
- Unfamiliarity with the IMG's home institution
- Budget implications
- Jeopardizing your program's reputation
- Potential for poor interactions with your patient population secondary to cultural or language differences
- Potential for poor interactions with your faculty or staff secondary to cultural or language differences
- Potential need for increased faculty guidance
- Inability to complete residency
- Leaving the U.S. after completing residency
- Visa Status

*After section 14, submit form*
